# Environmental Management Breadth, Environmental Management Depth, and Manufacturing Performance

**DOI:** 10.3390/ijerph16234628

**Published:** 2019-11-21

**Authors:** Qiang Zhang, Yuan Ma, Qiyue Yin

**Affiliations:** College of Economics and Management, Shandong University of Science and Technology, Qingdao 266590, China; zhangqiang3637@163.com (Q.Z.); may@sdust.edu.cn (Y.M.)

**Keywords:** environmental management breadth, environmental management depth, manufacturing performance, moderating effect

## Abstract

According to the number of environmental management practices implemented by the firm and the degree of integration of environmental management with other functions of the firm, this study divides environmental management into two dimensions: environmental management breadth and environmental management depth. We argue that the impact of environmental management breadth on manufacturing performance is moderated by environmental management depth. A survey data including 225 Chinese manufacturing firms is used to test the hypotheses. Results show that there is an inverted U-shaped relationship between environmental management breadth and manufacturing performance; the impact of environmental management depth is positive; meanwhile, environmental management depth moderates the relationship between environmental management breadth and manufacturing performance.

## 1. Introduction

There is a growing awareness by both public bodies and civil society toward environmental issues [1]. In response to environmental protection pressures, firms have adopted many environmental management (EM) measures [2]. There has been abundant research on the relationship between EM and manufacturing performance (MP) because MP is generally considered to be the main source of competitive advantage [3,4], but no consistent conclusions have been reached [5,6,7,8,9,10,11,12,13,14]. Some scholars believe that EM increases production and operating costs and reduces MP [5,6,7]. However, other studies have shown that EM can improve the working condition and employees’ health, enhance employee satisfaction, and thus promote firms’ MP [8,9].

EM is the management practices adopted by firms to monitor and control the impact of business activities on the natural environment [11]. These practices mainly include: (1) collecting environmental information; (2) developing environmental solutions; (3) handling environmental issues in compliance with the law; (4) conducting training and communication [11,15,16,17]. As a complex management activity, EM includes not only specific pollution prevention and control measures but also coordination and cooperation between the environmental department and other departments within firms, and the balance between environmental goals and other corporate goals [10,11,12]. EM practices differ in terms of resources required, and the impact on MP will be different [13,14,18,19]. 

Although some scholars recognize the complexity of environmental issues and emphasize effective and substantive EM, the extant research in this domain still regards EM as a whole, ignoring the internal differences of EM among different firms and drawing inconsistent conclusions. In order to further explore the influence mechanism of EM on MP, this paper divides EM into two dimensions: environmental management breadth (EMB) and environmental management depth (EMD) and studies their impacts on MP. EMB refers to the diversified EM practices implemented by firms; EMD refers to the level of coordination and cooperation between EM and other functions of the firm. The abbreviations of the nomenclatures can be seen in Appendix Table A1.

The paper makes several potential academic contributions. First, most studies regard EM as a holistic management activity. This study divides EM into two dimensions, EMB and EMD, and reveals the relationship between EM and MP in depth. Second, although previous studies generally believe that there is a linear relationship between EM practices and MP, this study theoretically further proves the existence of a nonlinear relationship between them. This study shows that the impact of EMB on MP presents an inverted U-shaped trend and that EMD moderates this relationship.

The remainder of the article is organized as follows. Section 2 reviews the literature and formulates hypotheses. The methodology used in the empirical study is described in Section 3, and then Section 4 presents the results of the analysis. Section 5 discusses the results of empirical analysis, with Section 6 presenting the conclusions.

## 2. Literature Review and Hypotheses Development

### 2.1. Environmental Management Breadth and Manufacturing Performance

Studies have shown that firms with EM systems typically have higher MP than their counterparts who have only developed simple EM solutions [20,21,22]. Therefore, firms tend to implement diversified EM practices and improve MP through comprehensive EM [18,21]. The improvement can be achieved through two conducts.

Firstly, EM can influence employees’ behavior, thereby increasing firms’ MP. Such EM practice as employee training programs can foster interpersonal interaction and employee engagement at work [8]. Training can make employees more efficient, and interpersonal relationships can help employees transfer knowledge and generate innovative ideas that increase MP [9,23]. In addition, interpersonal communication can promote employees’ job satisfaction and enthusiasm, thereby increasing MP [24,25]. EM can also improve the social image of the firm and enhance employees’ sense of identity with the firm [24], then improve employees’ morale and increase firms’ MP.

Secondly, EM can reduce the cost of firms and thus improve their MP. This includes making the relationship between firms and external stakeholders (government, environmental organizations, media, community, etc.) more harmonious, and reducing related risk management costs [24]; improving the use efficiency of resources, reducing the cost of raw materials and energy [8,26,27]; reducing financing costs because firms with proactive EM are more likely to gain the trust of banks or investors [24]; reducing labor costs because good environmental performance can reduce employee illness and absenteeism, and help firms attract and retain talents [28,29].

Reasonable EM practices can promote MP of firms, but when EM practices are too diversified to improve the environmental reputation and legitimacy of firms beyond the level that firms can afford, EM may damage firms’ MP [2,18,30]. Although most firms achieve initial cost savings by making up for existing inefficiency and resource waste through EM activities, EM costs will increase once the "harvest period" is passed [27,31]. As EM practices increase, the difficulty for firms to gain from EM increases. The cost may exceed the benefits brought by EM activities. In this case, it is difficult to improve and even hinder the MP of firms [21,31]. We therefore propose the following hypothesis:

**Hypotheses** **1 (H1).** *There is an inverted U-shaped relationship between EMB and MP*.

### 2.2. Environmental Management Depth and Manufacturing Performance

With the deepening of EM research, scholars have begun to realize that EM practices are not independent; they need coordination among technology, resources, and the strategy of the firm [13,14]. Margerum and Born [10] introduced the concept of “integration of environmental management” to solve complex environmental problems through comprehensive, interactive, and efficient methods. EM is an extensive and cross-functional challenge. Only by involving various functional departments devoting to EM can firms find comprehensive solutions to environmental problems [32,33,34,35,36].

First, the participation of the environment department, R&D department, and production department can ensure that the firm’s products meet environmental, quality, and health and safety requirements, which is conducive to reducing internal conflicts and saving production costs and improving the production efficiency [37]. Support from functional departments can provide corresponding information and employee motivation for EM [38,39]. In addition, information sharing and cooperation between functional departments and the environmental department are also helpful in accelerating the development of environmentally friendly products [40,41,42].

Second, the integration between different departments facilitates the transfer of successful management routines to EM and provides ideas for solving environmental problems (for example, applying quality management experience to EM) [43]. Through integration, firms can use internal resources, implement and supervise EM across departments effectively, and improve firms’ MP [37,44,45]. 

Therefore, by strengthening the integration of EM and other functions, and improving EMD, firms can obtain tacit capabilities and strategic resources, which are difficult to imitate [37,46,47]. These capabilities and resources not only help firms improve environmental performance, but also promote the development of MP and achieve competitive advantage [48,49,50]. Therefore, we propose the following hypothesis:

**Hypotheses** **2 (H2).** *EMD has a positive relationship with MP*.

### 2.3. The Moderating Role of Environmental Management Depth

As EM practices increase, firms must learn how to coordinate different EM activities and make complex configurations in terms of employees, resources, and technology [2,19,51,52]. EM integration can reduce the inefficiency caused by inconsistent departmental objectives in the manufacturing process [53] and create unique resources for firms to improve their competitive advantages [54,55]. The integration of EM in firms can reduce the cost of EM in terms of compliance, communication, and coordination of objectives. Therefore, the integration of EM can offset the cost of EM to some extent [20]. Integration of EM with other functions can also benefit firms in scale economy, complementary and cross-functional coordination, and ensure that firms meet or exceed environmental, quality, and health and safety standards [55,56,57]. Molina-Azorín et al. [52] pointed out that integration between different departments of the firm has a positive impact on economic performance, indicating that integration can effectively play a role in moderating the relationship between EM and economic performance. Therefore, the EMD, that is, the integration of EM and other functions, can moderate the relationship between EMB and MP. Hence the following hypothesis can be proposed:

**Hypotheses** **3 (H3).** *EMD positively moderates the relationship between EMB and MP*.

**Figure 1:**
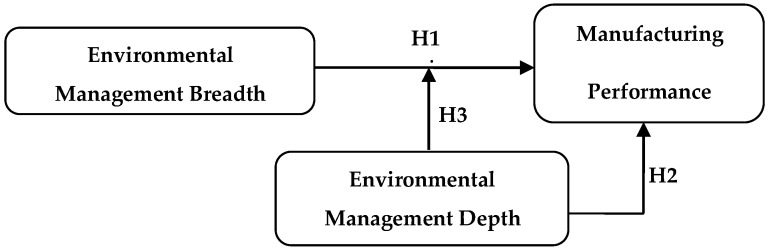
Theoretical framework.

The theoretical framework of this paper is shown in Figure 1.

## 3. Methodology

### 3.1. Sample

The sample of this study is the manufacturing industry in Shandong Province, China. China is experiencing unprecedented economic, social, and environmental changes. China’s development model has long placed economic growth above environmental protection, making it one of the most polluted countries in the world [58,59]. However, since the 11th Five Year Plan (2006–2010), the priorities of China’s economic development and environmental protection have begun to change. The government has placed more emphases on resource conservation, environmental protection, and climate change [60]. In the 13th Five Year Plan, the Chinese government clearly pointed out that it is necessary to achieve a win-win situation for economic development and environmental improvement. Shandong Province is one of the most developed provinces in China, with the third largest economy scale in the country and the important manufacturing province in China [61]. Therefore, Shandong Province has long faced serious environmental problems. In order to improve environmental quality and achieve sustainable development, the provincial government has enacted and implemented strict environmental regulations, and the manufacturing industry in Shandong Province is facing enormous environmental pressure [62]. Therefore, in this study, manufacturing firms in Shandong Province are suitable for investigation.

According to the list provided by the local business associations, a total of 600 questionnaires were issued in this study. The questionnaires were asked to be answered by the general manager of the firm or the environmental manager or department manager with relevant knowledge. A total of 256 questionnaires were collected, and 225 valid questionnaires were obtained after removing invalid questionnaires, with an effective recovery rate of 37.5%. The demographic characteristics of the informants and sample firms are shown in Table 1.

In order to test the non-respondent bias, we divided the samples into two groups according to the recycling order. The t-test results show that there are no significant differences between the two groups in terms of firm age, scale, and ownership. Therefore, there is no non-respondent bias in this study [63].

### 3.2. Measurement 

The scales used in this study refer to the maturity scales of foreign studies, and a two-way translation method was adopted in the questionnaire design. After pre-investigation and expert discussion, each item was carefully refined and corrected to ensure the accuracy of the description.

The dependent variable in this study is EMB. EMB is measured by the number of EM practices implemented by the firm. Drawing on the research of Khanna and Anton [21], 12 EM practices were contained in the questionnaire. These practices indicate the firm’s multifaceted efforts that are proactive and anticipative in orientation, targeted towards improving environmental performance [21]. The adoption of each practice is represented by a dummy variable, 1 = yes; 0 = no. Therefore, we measure EMB by summing these dummy variables.

EMD means the linkage of goals and activities related to EM with core managerial processes and functions in other areas that are of strategic relevance to the firm, namely its corporate strategy, quality management, Health & Safety, and social issues [44,49]. So, a scale including 4 items was used to measure EMD (i.e., the integration of EM with other functions of the firm). Informants scored through the Likert five-point scale (from “very disagree” to “completely agree”). The specific items of EMB and EMD can be seen in Appendix Table A2.

MP is measured by the ratio of total output to operation input [64]. Drawing on the study by Lannelongue et al. [3], this study uses annual average sales per employees after natural logarithm as a measure.

This study set four control variables: firm size (the natural logarithm of the employees’ number), firm age (the natural logarithm of the age of the firm), types of industry (take the manufacturing double-digit code issued by the China Securities Regulatory Commission), and ownership (state-owned = 1, non-state-owned = 0).

## 4. Research Result

### 4.1. Reliability, Validity, and Descriptive Statistics

The factor analysis of the EMD scale produced only one factor, thus verifying the one-dimensionality of the variable. Meanwhile, the Cronbach’s α coefficient of this scale is 0.774, indicating that it has a high reliability.

Table 2 shows descriptive statistics and correlations between study variables. The significant correlation between the variables provides the basis for further analysis.

### 4.2. Regression Results

To avoid potential multicollinearity problems, we first centralized the variables related to interaction and square terms and then tested the research hypotheses with SPSS 25.0 software (SPSS Inc., Chicago, IL, USA). Table 3 shows the specific regression analysis results.

The impact of EMB on MP is shown in Model 2. The result of Model 2 shows that the coefficient of the squared term of EMB on MP is negative (β = −0.306, *p* < 0.001), indicating that there is an inverted U-shaped relationship between EMB and MP. Therefore, hypothesis H1 is supported. The result of Model 3 shows that the coefficient of EMD on MP is positive (β = 0.444, *p* < 0.001), indicating that EMD can improve firms’ MP. Hypothesis H2 is supported.

**Figure 2:**
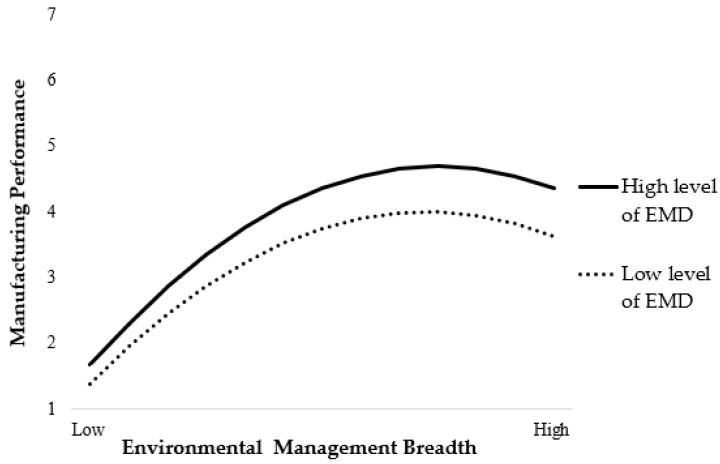
Interaction Graph.

Finally, the moderating role of EMD is tested. Model 4 shows that the coefficient of the interaction term between EMD and the square of EMB is significant (β = −0.291, *p* < 0.001), indicating that EMD significantly moderates the inverse U-shaped relationship between EMB and MP. Hypothesis 3 is supported. Following the procedure proposed by Aiken and West [65], we took two values for the variables of EMB and EMD, respectively: mean minus one standard deviation, and mean plus one standard deviation. The moderating effect is shown in Figure 2. It can be seen from the figure that the impact of EMD on MP is more obvious for firms with a high degree of EMD.

## 5. Discussion

This study empirically tested the relationship between EMB, EMD, and MP. Here, we discuss the results in detail.

First, it is found that there is an inverted U-shaped relationship between EMB and MP. Appropriate adoption of EM practices in firms is conducive to improving MP, and excessive pursuit of comprehensiveness of EM practices will hinder MP. When EMB matches the resources and capabilities of the firm, EM can help firms achieve better human resource management and cost reduction and increase MP. To be specific, employees are proud of the firm’s good environmental reputation and perform better at work [24]; relevant EM training improves employees’ work skills and promotes interpersonal communication and knowledge dissemination among employees [24,25]; and EM practices reduce the cost of the firm (including the management of risks and of dealings with external stakeholders, the cost of material, energy and services, the cost of capital, and the cost of labor) [25,26,27,28]. Lucchi also confirmed this result in the study of the environmental and energy quality in museum buildings [66]. However, the implementation of EM activities by firms is a “double-edged sword”. Various EM practices are coordinated and complementary with each other. Too many EM practices will occupy a large amount of resources, which will put a heavy burden on the firm, leading to diminishing marginal benefits and reducing MP. 

Second, EMD has a positive effect on MP. Our research consolidates the standpoints of Wagner [49] and Hart and Dowell [50]. EM is an extensive and cross-functional challenge that can only be fully effective if it is integrated and interacted with other functional departments of the firm [43]. The integration of EM with R&D, production and other functions is conducive to the information sharing among departments, and the formulation of product standards that conform to the environment, quality, health, and safety at the same time, thereby reducing internal conflicts and improving MP [37,38,39,40,41,42]. Therefore, strengthening the integration of EM and other functions will help firms achieve unique competitive advantages and increase MP.

Third, this study also explored the moderating role of EMD. As EMD increases, the impact of EMB on MP is more obvious. The integration of EM and other functions can coordinate the objectives of different departments and facilitate the optimal configuration of personnel, resources, and technology [53,54,55,56,57]. In addition, through integration, EM can absorb the management experience of other functional departments and improve the efficiency of EM practices. Therefore, the benefits of integrating EM with other functions can offset the cost of EM practices, and can also control the relationship between EM and economic performance through internal coordination. 

## 6. Conclusions and Implications

### 6.1. Conclusions

As environmental regulations become more stringent and stakeholders’ environmental awareness continues to increase, EM is becoming more and more important for firms. Although many scholars have studied the relationship between EM and firm economic performance, the research conclusion is still not clear. To further explore the impact of EM on firms’ MP, this study divides EM into two dimensions: EMB and EMD. EMB refers to the number of EM practices implemented by firms; EMD refers to the degree of integration of EM practices with other functions of the firm. Using the survey data from 225 firms, the relationships between EMB, EMD, and MP were tested empirically. Results show that there is an inverted U-shaped relationship between EMB and MP, EMD has a positive effect on MP, and EMD moderates the relationship between EMB and MP. 

### 6.2. Implications

The results of this study can provide some inspiration for business managers. For firms, EM practices are not the more, the better. In order to meet the requirements of environmental regulations and stakeholder expectations, improve the "legitimacy" of firms, firms have taken a large number of EM practices. Implementing appropriate EM practices will help firms achieve better MP, but excessive environmental protection measures will impose a heavy burden on the firm and damage the firm’s economic interests. Therefore, more EM practices are not always better for firms. Firms should not only pay attention to the "quantity" of EM but also pay attention to the "quality". Managers need to balance the relationship between EM and economic development and carefully consider the EM practices that need to be implemented. On the other hand, considering that EMD is beneficial to MP and it moderates the relationship between EMB and MP, firms should make efforts to improve EMD, promote the coordination and interaction of EM and other functions, and integrate EM strategies and objectives into other functional departments to improve the efficiency and effectiveness of EM. Achieving this balance is not easy, because it requires managers to have rich management experience, and other departments to support and cooperate with the EM department.

## 7. Limitations and Future Research

Our research is not without its limitations, which should also be seen as opportunities and challenges for future investigations. First, the study used cross-sectional data, which could be further tested by tracking data or longitudinal studies. Second, this study only selected the manufacturing industry as a research sample, and the industries with great environmental pressure are not only manufacturing, for example, more and more service companies are also involved in EM [67]. Future research could increase the sample size and cover more industries and improve the universal applicability of research results. In addition, how to maintain the best EM practices is also a problem that needs further research.

## Figures and Tables

**Table 1:** Demographic traits of informants and sample firms.

Informants	Percentage (%)	Sample Firms	Percentage (%)
Gender		Number of employees	
Male	77.8	Less than 300	43.1
Female	22.2	300–1000	32.0
Departments		More than 1000	24.9
Environmental	24	Ownership	
Management	45.3	State-owned	39.5
Others	30.7	Non-state-owned	60.5
Positions		Age	
Junior manager	31.1	1–5	14.2
Senior manager	45.3	6–10	28.9
Top manager	23.6	More than 11	56.9
Tenure			
1–5	34.2		
6–10	21.8		
More than 11	44		

**Table 2:** Descriptive statistics and correlation analysis.

Variables	MP	OWN	AGE	IND	SIZE	EMB	EMD
MP	1.000						
OWN	−0.197 **	1.000					
AGE	0.173 **	0.049	1.000				
IND	−0.424 **	0.140 *	0.127	1.000			
SIZE	−0.130 **	0.276 **	0.493 **	0.181 **	1.000		
EMB	0.461 **	0.061	−0.212 **	−0.173 **	0.029	1.000	
EMD	0.335 **	−0.027	−0.289 **	−0.385 **	−0.095	0.487 **	1.000
Mean	3.163	0.300	2.872	4.080	5.936	8.73	3.980
S.D.	1.074	0.458	0.564	2.166	1.031	1.902	0.609

Note: * *p* < 0.05, ** *p* < 0.01 (2-tailed). MP = manufacturing performance, EMB = environmental management breadth, EMD = environmental management depth, OWN = ownership, AGE = firm age, IND = industry, SIZE = firm size, S.D. = standard deviation.

**Table 3:** Results of regression analysis.

Variables	MP			
	Model 1	Model 2	Model 3	Model 4
OWN	−0.104	−0.174 ***	−0.107 *	−0.175 ***
AGE	0.322 ***	0.557 ***	0.455 ***	0.480 ***
IND	−0.417 ***	−0.252 ***	−0.253 ***	−0.247 ***
SIZE	−0.184 **	−0.285 ***	−0.237 ***	−0.186 ***
EMB		0.647 ***		0.766 ***
EMB^2^		−0.306 ***		−0.507 ***
EMD			0.444 ***	0.098 **
EMD × EMB				0.113 *
EMD × EMB^2^				−0.291 ***
R^2^	0.277	0.852	0.431	0.879
Adjusted R^2^	0.263	0.848	0.418	0.874
F	20.755 ***	205.733 ***	32.684 ***	171.450 ***

Note: * *p* < 0.05, ** *p* < 0.01, *** *p* < 0.001, data in this table are standardized coefficients. MP = manufacturing performance, OWN = ownership, AGE = firm age, IND = industry, SIZE = firm size, EMB = environmental management breadth, EMD = environmental management depth. EMB^2^ = square term of environmental management breadth

## Appendix A

**Table A1:** Nomenclatures and abbreviations.

Nomenclatures	Abbreviations
Environmental management	EM
Environmental management breadth	EMB
Environmental management depth	EMD
Manufacturing performance	MP

## Appendix B

**Table A2:** Survey Items.

Please judge the following statements based on your company’s actual situation. 1 = “Yes”, 0 = “No”.		
Environmental Management Breadth	A1 Company has a dedicated environmental management department.	Khanna & Anton, 2002
	A2 Company has a formal written policy and codes of conduct on environmental issues.	
	A3 Company applies uniform standards to environmental practices worldwide.	
	A4 Company purchases insurance to meet unexpected environmental liabilities.	
	A5 Company applies total quality management philosophy to environmental management.	
	A6 Company provides incentive compensation to employees whose efforts lead to the achievement of specific environmental goals.	
	A7 Company conducts audits to assess compliance with environmental regulations.	
	A8 Company evaluates its environmental risks when selecting its suppliers.	
	A9 Company evaluates its environmental risks when selecting its partners.	
	A10 Company evaluates its environmental risks when selecting its clients.	
	A11 Company regularly releases reports about its environmental performance and activities.	
	A12 Company sets aside funds to cover the costs of penalties for environmental violation or remediation activities.	
Please judge the following statements based on your company’s actual situation. 1 = “very disagree”, 2 = “disagree”, 3 = “unsure”, 4 = “agree”, 5 = “strongly agree”.		
Environmental Management Depth	B1 The company’s environmental management activities are fully integrated with activities related to “quality assurance”.	Wagner, 2007, 2015
	B2 The company’s environmental management activities are fully integrated with activities related to “social issues”.	
	B3 The company’s environmental management activities are fully integrated with activities related to “health and safety”.	
	B4 The company’s environmental management activities are fully integrated with activities related to “corporate strategy”.	
